# Modulation of miR‐204 and miR‐375 Expression by Flavonoid Chrysin Treatment During Melphalan‐Induced Histopathological Damage in Adult Rat Skin and Hair Tissue

**DOI:** 10.1002/fsn3.71946

**Published:** 2026-05-24

**Authors:** Maryam Moghimian, Zahra Parsamanesh, Zahra Rajabzadeh, Negar Shafaei‐Bajestani, Malihe Soltani

**Affiliations:** ^1^ Department of Physiology, Faculty of Medicine Gonabad University of Medical Sciences Gonabad Iran; ^2^ Student Research Committee Gonabad University of Medical Sciences Gonabad Iran; ^3^ Department of Pharmacology, Faculty of Medicine Gonabad University of Medical Sciences Gonabad Iran; ^4^ Department of Anatomy, School of Medicine Gonabad University of Medical Sciences Gonabad Iran

**Keywords:** chrysin, flavonoid, hair, melphalan, nutraceutical, skin

## Abstract

Chemotherapy‐induced skin and hair follicle damage, driven in part by miRNAs such as miR‐204 and miR‐375 that regulate apoptosis and autophagy after alkylating agent exposure, significantly impacts patient quality of life. While chrysin—a common food‐derived flavonoid and nutritional bioactive with antioxidant and cytoprotective properties—has shown potential to modulate miRNA activity, its role in melphalan‐induced injury and associated miRNA expression remains unclear. This study investigated the effects of melphalan on skin and hair follicle integrity in rats, focusing on miR‐204 and miR‐375 expression, and evaluated the protective potential of chrysin treatment. Forty‐eight male Wistar rats were divided into six groups receiving melphalan (1.5 mg/kg), chrysin (50 or 75 mg/kg), or their combination for 21 days. Dorsal skin samples were analyzed using histological and morphometric assessments. Expression levels of apoptosis‐related genes, autophagy markers, and miR‐204/miR‐375 were quantified by RT‐qPCR. Melphalan significantly reduced epidermal thickness and anagen‐phase hair follicles while increasing apoptosis markers (BAX, caspase‐3) and suppressing autophagy‐related genes. It also markedly upregulated miR‐204 and miR‐375. Chrysin co‐administration, particularly at 75 mg/kg, correlates with restored epidermal thickness, improved hair follicle cycling, enhanced autophagy marker expression, suppressed apoptosis, and significantly downregulated miR‐204 and miR‐375. Chrysin reduces melphalan‐induced skin and hair follicle damage, possibly correlating with modulation of apoptosis and autophagy pathways and suppression of miR‐204 and miR‐375. These findings suggest chrysin as a promising nutraceutical candidate and supportive dietary strategy to help reduce chemotherapy‐associated dermatological side effects.

## Graphical abstract

Rats were treated with melphalan (1.5 mg/kg) with or without chrysin (50 or 75 mg/kg) for 21 days. Melphalan reduced epidermal thickness, disrupted the hair follicle cycle, increased apoptosis, suppressed autophagy, and upregulated miR‐204/miR‐375. Chrysin, particularly at 75 mg/kg, reversed these effects by restoring skin structure, enhancing autophagy, reducing apoptosis, and downregulating miR‐204/miR‐375.

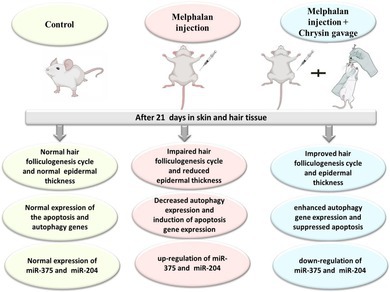

## 1 Introduction

In recent years, increasing attention has been directed toward understanding the regulatory roles of microRNAs (miRNAs) in cellular processes such as proliferation, differentiation, apoptosis, and autophagy within various tissues, including the skin and hair follicles (Gerasymchuk et al. 2020). MiRNAs are short, non‐coding RNA molecules that function post‐transcriptionally to modulate gene expression, playing critical roles in tissue homeostasis and responses to external stressors (Wang et al. 2024; Zhai et al. 2021). Among the numerous miRNAs, miR‐204 and miR‐375 have emerged as key regulators of apoptosis and autophagy. These miRNAs have been shown to act as autophagy inhibitors and apoptosis inducers during tissue damage (Fallahian et al. 2025; Wang and Yang 2023; Wei et al. 2021; Yao et al. 2023), particularly under the influence of cytotoxic agents, including chemotherapeutics. However, despite the well‐documented involvement of miRNAs in tissue responses to injury, the expression patterns and biological implications of miR‐204 and miR‐375 in skin and hair tissues subjected to chemotherapy remain poorly understood.

Alkylating agents, a cornerstone in many chemotherapy regimens, inflict genotoxic stress by covalently modifying DNA, leading to impaired replication and cell cycle arrest (van den Boogaard et al. 2022). Melphalan, a bifunctional alkylating agent of the nitrogen mustard class, is widely employed in the treatment of hematological and solid malignancies, including multiple myeloma, ovarian carcinoma, and malignant melanoma (Dixit et al. 2022). While melphalan is effective in targeting rapidly proliferating cancer cells, its cytotoxicity also impacts healthy tissues with high mitotic rates, such as the epidermis and hair follicles (Alrifaie 2023). These off‐target effects commonly manifest as histopathological changes, including thinning of the epidermis, depletion of follicular stem cell populations, and compromised tissue regeneration (Kapoor et al. 2025). Clinically, such adverse reactions contribute to dermatological complications and alopecia, which significantly reduce patients' quality of life during and after chemotherapy (Almeida et al. 2023).

Given the vulnerability of skin and hair tissues to chemotherapeutic‐induced damage, there is a growing interest in identifying agents that can ameliorate such side effects without compromising anticancer efficacy. Natural flavonoids, particularly chrysin, have emerged as promising candidates due to their diverse bioactive properties (Mohammed et al. 2023). Chrysin (5,7‐dihydroxy‐2‐phenyl‐4H‐chromen‐4‐one) is a naturally occurring dietary flavonoid commonly found in honey, propolis, and various plant‐based foods and is increasingly studied as a bioactive compound in the context of functional foods and nutraceuticals. Although its habitual dietary intake is relatively low, concentrated forms of chrysin are available in nutraceutical preparations, raising interest in its potential role as a supportive dietary agent under conditions of physiological stress (Kurkiewicz et al. 2025; Rahmani and Khan 2025).

From a nutritional perspective, the relevance of chrysin is also influenced by its pharmacokinetic profile. Previous studies indicate that chrysin exhibits relatively low oral bioavailability due to rapid metabolism and limited absorption. However, emerging formulation strategies (e.g., nanoformulations, co‐administration with bioavailability enhancers) may improve its systemic availability, supporting its plausibility as a functional food ingredient or nutraceutical candidate (Jeong et al. 2025; Rahmani and Khan 2025). It is known for its strong antioxidant, anti‐inflammatory, and autophagy‐modulating activities (Deng et al. 2025; Mohammed et al. 2023). Previous research suggests that chrysin can protect cells and tissues from oxidative and chemical insults, reduce apoptotic cell death, regulate key miRNAs, and promote tissue regeneration (Ebadi et al. 2025). Its protective properties have been shown to counteract the detrimental effects of chemotherapeutics like Cisplatin and doxorubicin (Mohamad et al. 2025; Ye et al. 2022). However, the potential for chrysin to mitigate melphalan‐induced histopathological damage and modulate miRNA activity in skin and hair tissues remains largely unexplored.

This study examined the expression of miR‐204 and miR‐375 in rat skin and hair follicles following melphalan‐induced damage and evaluated whether treatment with the flavonoid chrysin can modulate these responses. By elucidating the protective effects of chrysin and its impact on miRNA expression, this research offers novel insights into the development of nutrition‐based supportive strategies and nutraceutical approaches aimed at preserving tissue integrity during chemotherapy.

## 2 Materials and Methods

### 2.1 Experimental Design and Animal Handling

The experiment involved 48 adult male Wistar rats, each weighing approximately 225 ± 25 g. All animals were housed in the animal care facility at Gonabad University of Medical Sciences, where they were maintained under standard environmental conditions—12‐h light/dark cycles, temperature controlled at 22°C ± 2°C, and with adequate airflow. The rats had unrestricted access to food and water throughout the adaptation and experimental periods. The entire study was performed in compliance with ethical standards for the care and use of laboratory animals, as governed by the guidelines established by the Ethics Committee of the Ministry of Health and Medical Education of the Islamic Republic of Iran.

Animals were randomly divided into six experimental groups, each containing eight rats, according to the treatment regimen with chrysin:

– Group 1 (Control): Normal saline injection along with corn oil gavage for 21 days.
– Group 2 (Mel): Administered melphalan intraperitoneally (1.5 mg/kg/day) for 21 days.
– Group 3 (Mel+Chr50): Treated with melphalan (1.5 mg/kg/day, i.p.) and chrysin (50 mg/kg/day, orally) for 21 days.
– Group 4 (Mel+ Chr75): Received melphalan (1.5 mg/kg/day, i.p.) and chrysin (75 mg/kg/day, orally) for 21 days.
– Group 5 (Chr50): Given chrysin alone (50 mg/kg/day, orally) for 21 days
– Group 6 (Chr75): Administered chrysin alone (75 mg/kg/day, orally) for 21 days.

The route and dosage of melphalan (1.5 mg/kg, i.p.) were based on prior literature (Panghal et al. 2023). Chrysin, dissolved in corn oil, was administered daily by gavage during the same treatment period (Ebadi et al. 2025). *Melphalan*: Manufactured by EXCELLA GmbH, Nürnberg Strasse 12, 90,537 Feucht, Germany. *Chrysin*: 5,7‐dehydroxyflavone (97%) SIGMA‐ALDRICH (Pcode: 101896550).

### 2.2 Anesthesia, Sacrifice, and Sampling Method

At 24 h after completion of the last dose, the animals were anesthetized using a ketamine (50 mg/kg) and xylazine (10 mg/kg) cocktail (Soltani et al. 2022). Following confirmation of deep anesthesia, dorsal skin samples were collected for molecular and histological evaluations. Subsequently, the animals were sacrificed in accordance with approved ethical procedures.

### 2.3 Histological and Morphometric Assessments

Histopathological damage was confirmed by blinded evaluation of H&E sections using criteria including epidermal necrosis, inflammatory infiltration, dermal edema or hemorrhage, and follicular dystrophy.

Also, epidermal thickness was measured quantitatively using ImageJ software (v1.53, NIH) following calibration with a stage micrometer. Thickness was defined as the perpendicular distance from the stratum basale to the outermost layer of the stratum corneum. In such a way that three random measurements were taken per field at 400× magnification, avoiding hair follicle openings and skin edges. A total of 10 fields per section were analyzed. Using ImageJ's straight‐line tool, measurements were performed in triplicate per field, and the mean value was recorded for that field. Values are expressed as micrometers (μm).

Hair Follicle Classification (Anagen/Catagen/Telogen): Follicle classification was performed according to established morphological criteria, with stage determination based on the following histological features: *Anagen*: Deep dermal/subcutaneous localization; presence of thickened, pigmented hair bulb; inner root sheath present; dermal papilla fully enclosed by matrix cells; high mitotic activity in bulb. *Catagen*: Shrinking hair bulb; condensed epithelial strands; apoptosis‐driven regression of the lower follicle; ascending follicle position; thickened, wrinkled basement membrane. *Telogen*: Club hair with trichilemmal keratinization; small, quiescent secondary germ cells; absence of inner root sheath; dermal papilla separated from epithelial cells; superficial dermal location.

### 2.4 Assessment Protocol

Sampling: For each mouse, three non‐consecutive sections (spaced at least 100 μm apart to avoid counting the same follicle twice) were analyzed.

Quantification: All hair follicles within the entire dermal depth of each section were classified (typically 30–50 follicles per section). The anagen‐to‐catagen ratio was calculated as: (Number of anagen follicles)/(Number of catagen follicles). Telogen follicles were recorded separately but excluded from this specific ratio calculation (Eid et al. 2025; Salah et al. 2025).

### 2.5 Molecular Investigations

Fresh dorsal skin tissue was homogenized after anesthesia to study gene expression via real‐time quantitative PCR (RT‐qPCR). The investigation focused on gene markers associated with autophagy (LC3β, Beclin‐1, Atg5, Atg7, Atg12), cell apoptosis (BAX, Bcl‐2, caspase‐3), and specific regulatory microRNAs (miR‐375 and miR‐204).

### 2.6 qRT‐PCR Protocol for mRNA Analysis

Total RNA was extracted using a commercial isolation kit (Yektatajiz, Iran). RNA quality was assessed using agarose gel electrophoresis. Subsequently, cDNA was synthesized from 1 μg of RNA using a reverse transcription kit (Yektatajiz, Iran). Real‐time PCR reactions were carried out in a 10 μL volume, composed of 1 μL of cDNA, 0.2 μM of forward and reverse primers, 5 μL of SYBR Green Master Mix (Ampliqon, Denmark), and 3.6 μL of nuclease‐free water using the StepOne Real‐Time PCR System (Applied Biosystems, USA). The thermal cycling conditions were: initial denaturation at 95°C for 10 min, followed by 40 cycles of 95°C for 15 s, 58°C for 30 s, and 72°C for 30 s. A melting curve analysis was included at the end. GAPDH was used as a reference gene, and relative expression levels were quantified via the 2^−ΔΔCT^ method (Roshanaee et al. 2022).

### 2.7 MicroRNA Expression Analysis Using Stem‐Loop RT‐qPCR

The isolation of total RNA for microRNA quantification followed the same protocol as mRNA extraction. Reverse transcription was carried out using stem‐loop primers specific for the target miRNAs. The reverse transcription reaction (10 μL total) consisted of 0.5 μL of reverse transcriptase (Thermo Scientific, USA), 2 μL of reaction buffer, 1 μL of stem‐loop primer, 1.5 μL of dNTP mix, 1 μL of RNA (~500 ng), and 4 μL of nuclease‐free water. The thermal profile included incubation at 16°C for 30 min, 42°C for 30 min, and termination at 82°C for 5 min. qPCR was conducted with RNU6 used as the internal housekeeping control for normalization (Saadatian et al. 2023).

Primer sequences used in RT‐PCR were as follows:

- *Bax (F primer: TTG CTA CAG GGT TTC ATC CA/R primer: GAG TAC CTG AAC CGG CAT CT)*,
- *Bcl*_*2*_ *(F primer: GCTACCGTCGTGACTTCGC/R primer: CCCCACCGAACTCAAAGAAGG)*,
- *Caspase3 (F primer: TGTGCTCCAGGCTTCCTTAATC/R primer: AGGCTTATGGGAAATGCTGGAC)*,
- *Beclin‐1 (F primer: CGAAAGGTGGTGGCAGAAAAC/R primer: ACTATATTCTCGCTGGTACTGAGC)*,
- *Lc3 β (F primer: TCAGTGAGAGCTGCCTCTGTC/R primer: AGCAGTGGGGATTTACACAGTG)*,
- *Atg5 (F primer: AGATCACAGTTCTGGGATGC/R primer: TCAGGCGGTAGAGATCGTAG)*,
- *Atg7 (F primer: TGTCTTGCAGCATCCTGAG/R primer: TCAAGAACTTTGGATGAACAGG)*,
- *Atg12 (F primer: GCACTCATCGACTTCATCAG/R primer: ACTGCCAAAACACTCATATAGAG)*,
- *GAPDH (F primer: GGTCTACATGTTCCAGTATGACTC/R primer: CATTTGATGTTAGCGGGATCTCG)*,
- *MIR‐375‐3P (F primer: GTCGTATCCAGTGCAGGGTCCGAGGTATTCGCACTGGATACGACTCACGC/R primer: CCCTTTGTTCGTTCGGCTC)*
- *MIR‐204‐5P (F primer: GTCGTATCCAGTGCAGGGTCCGAGGTATTCGCACTGGATACGACGGCAT/R primer: CCCCTTCCCTTTGTCATCCT)*,
- *U6 (F primer: GTCGTATCCAGTGCAGGGTCCGAGGTATTCGCACTGGATACGACAAAAAT/R primer: GCTTCGGCAGCACATATACTAAAAT)*.

### 2.8 Statistical Analysis

Data were analyzed using GraphPad Prism version 8. Values are reported as mean ± standard error of the mean (SEM). The assumption of data normality was validated using the Kolmogorov–Smirnov test. Statistical differences between groups were assessed using one‐way ANOVA followed by Tukey's post hoc test for multiple comparisons. A *p*‐value of < 0.05 was considered statistically significant.

## 3 Results

### 3.1 Histological Findings

**FIGURE 1:**
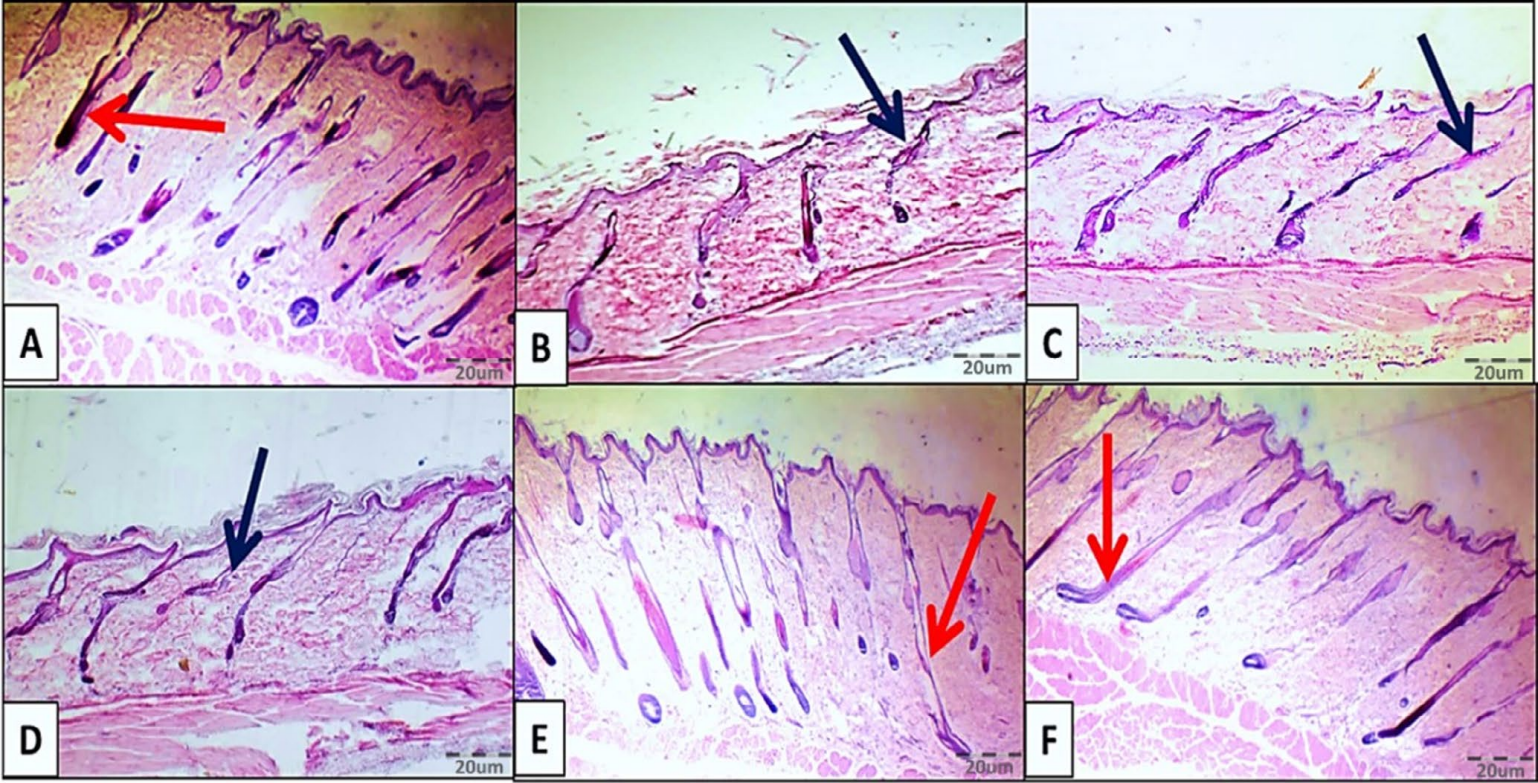
H&E staining images of adult rat skin tissue following melphalan administration and chrysin treatment in all study groups. Red arrows indicate the anagen phase of hair follicles. Black arrows indicate the catagen phase of hair. (A) Control group, (B) Mel group, (C) Mel +Chr50 group, (D) Mel +Chr75 group, (E) Chr50 group, (F) Chr75 group.

**FIGURE 2:**
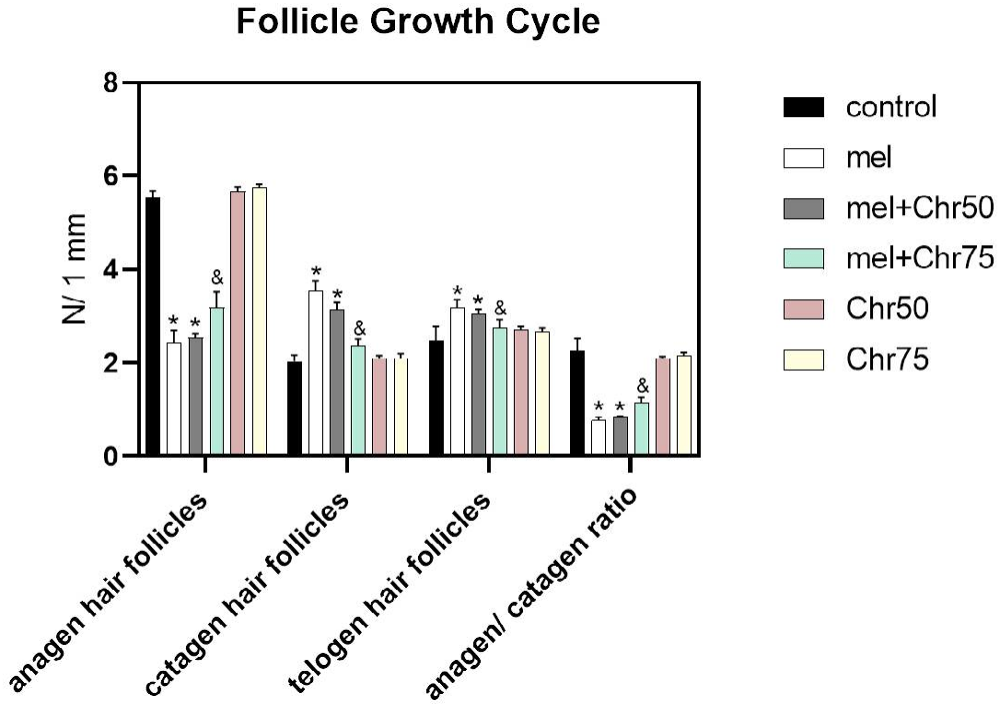
Comparison of histological analysis of hair follicle types in the growth phase (anagen), regression phase (catagen), and resting phase (telogen) of the folliculogenic cycle following melphalan and chrysin treatment in all study groups. The number of follicle types per millimeter was counted. Values are mean ± SE. *p* < 0.*05* are significant, *p < 0.05 versus Control, ^&^ *p* < 0.05 versus Mel group. M: Melphalan group, M + C50: Melphalan + Chrysin50 group, M + C75: Melphalan + Chrysin75 group, C50: Chrysin50 group, C75: Chrysin75 group.

The study evaluated the hair folliculogenesis cycle by quantifying follicles in the anagen (growth), catagen (regression), and telogen (resting) phases. Comparison with the control group revealed that melphalan administration (Mel group) caused a significant reduction in the mean number of anagen follicles, concurrently increasing the count of follicles in the catagen and telogen phases. However, co‐administration of chrysin at the higher dosage (Mel + Chr75) reversed this effect, yielding a significant rise in anagen follicles and a reduction in catagen and telogen counts compared to the Mel group. While the Mel + Chr50 group displayed similar restorative trends, these changes did not reach statistical significance (Figures 1 and 2).

**FIGURE 3:**
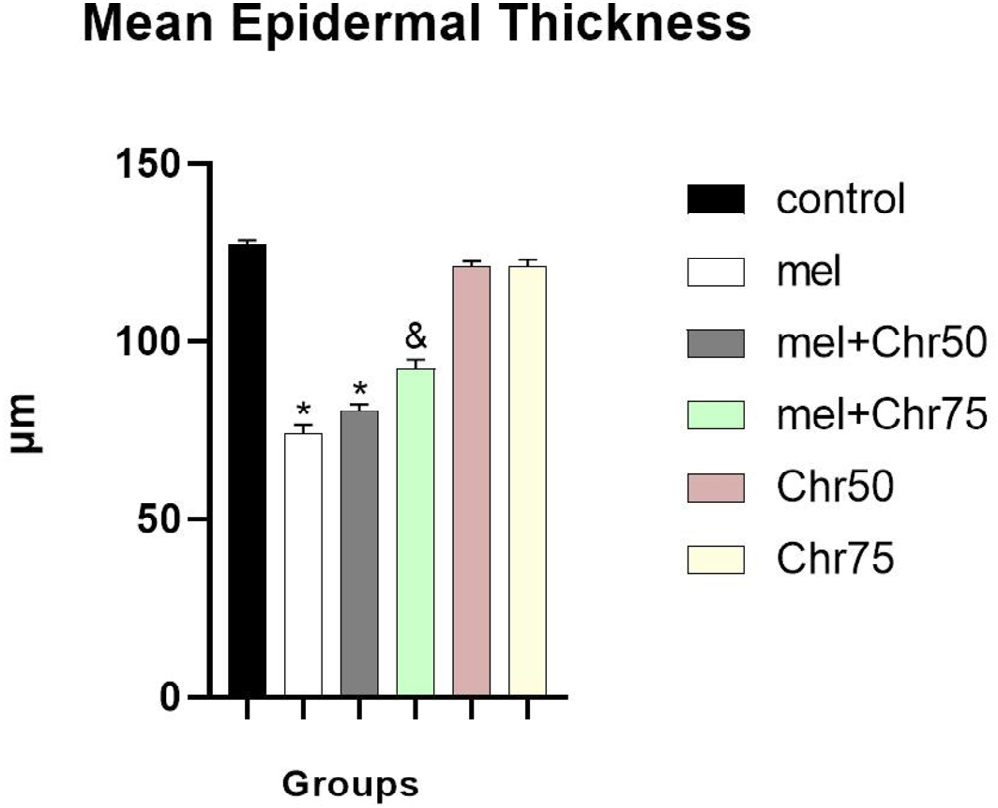
Comparison of histological analysis of skin epithelial thickness following melphalan and chrysin treatment in all study groups. Values are mean ± SE. *p* < 0.05 are significant, *p < 0.05 versus Control, ^&^ *p* < 0.05 versus cM group. Mel: Melphalan group, Mel +Chr50: Melphalan +Chrysin50 group, Mel +Chr75: Melphalan +Chrysin75 group, Chr50: Chrysin50 group, Chr75: Chrysin75 group.

Regarding epidermal morphometry, the Mel group exhibited a significantly reduced epithelial height compared to controls. Treatment with 75 mg/kg of chrysin significantly restored epidermal thickness relative to the melphalan‐only group. Although the Mel + Chr50 group showed a pattern of increased thickness, the improvement was not statistically significant (Figure 3).

### 3.2 Apoptotic and Anti‐Apoptotic Gene Expression

Analysis of pro‐apoptotic markers, specifically Caspase‐3, BAX, and BAX/BCL2 ratio indicated that melphalan treatment significantly upregulated these genes compared to the control group. Intervention with chrysin at 75 mg/kg (Mel + Chr75) significantly suppressed the expression of these markers relative to the Mel group. Conversely, the Mel + Chr50 group continued to exhibit elevated expression levels compared to controls.

**FIGURE 4:**
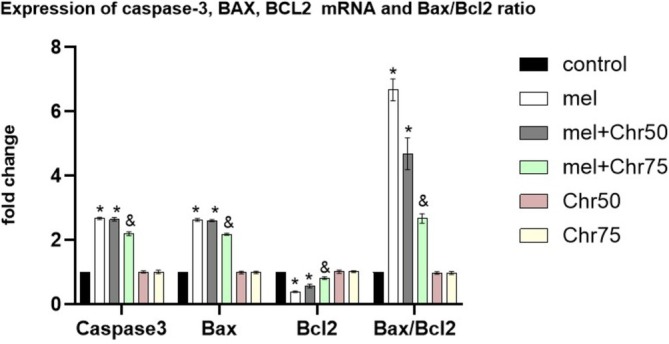
Real‐time analysis of caspase‐3, BAX, BCL2 genes as apoptosis markers in adult rat skin tissue following melphalan administration and chrysin treatment in all study groups. Values are mean ± SE. *p* < 0.*05* are significant, *p < 0.05 versus Control, ^&^ *p* < 0.05 versus Mel group.: Elphalan group, +Chr50: Melphalan +Chrysin50 group, +Chr75: Melphalan +Chrysin75 group, Chr50: Chrysin50 group, Chr75: Chrysin75 group.

In terms of the anti‐apoptotic marker BCL‐2, the Mel group showed a significant downregulation of gene expression relative to controls. While the Mel + Chr75 group demonstrated a significant recovery in BCL‐2 levels compared to the Mel group, BCL‐2 expression remained significantly suppressed in the Mel + Chr50 group when compared to the control baseline (Figure 4).

### 3.3 Autophagy Markers and MicroRNA Profiling

**FIGURE 5:**
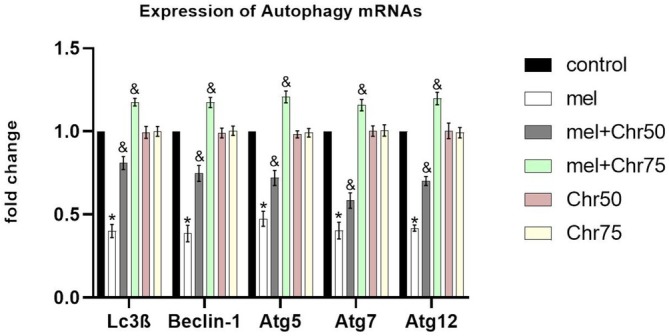
Real‐time analysis of LC3β, Beclin‐1, and Atg5, 7, 12 genes as markers of autophagy in adult rat skin tissue following melphalan administration and chrysin treatment in all study groups. Values are mean ± SE. *p* < 0.05 are significant, *p < 0.05 versus Control, ^&^ *p* < 0.05 versus cMEL group. mel: Melphalan group, mel+Chr50: Melphalan +Chrysin50 group, mel+Chr75: Melphalan +Chrysin75 group, Chr50: Chrysin50 group, Chr75: Chrysin75 group.

The expression of autophagy‐related genes—LC3β, Beclin‐1, and the Atg5, 7, 12 complexes—was significantly inhibited in the Mel group compared to controls. Notably, chrysin treatment at both tested dosages (Mel + Chr50 and Mel + Chr75) resulted in a significant upregulation of these autophagy markers compared to the melphalan‐treated group (Figure 5).

**FIGURE 6:**
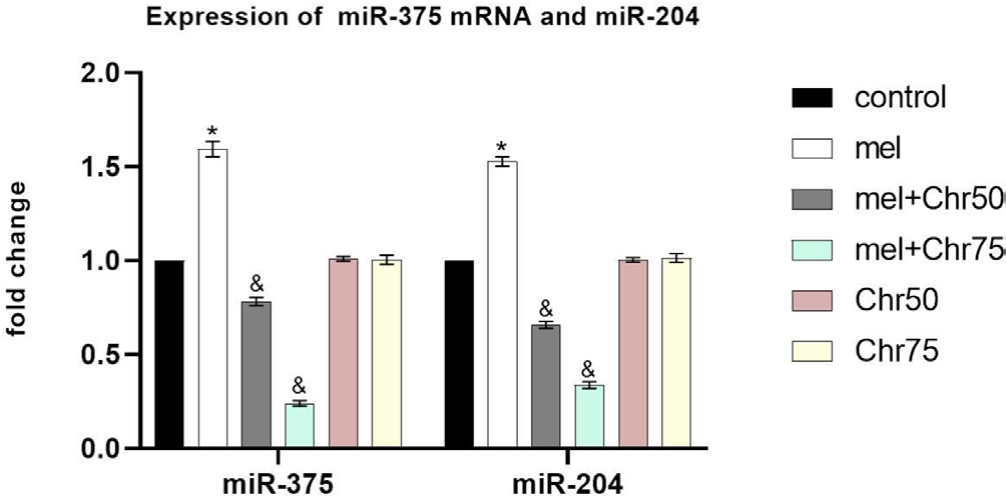
Relative expression of miR‐375 and miR‐204 in adult rat skin. QRT‐PCR analysis of miR‐375 and miR‐204 levels in skin tissue following melphalan administration and chrysin treatment in all study groups. Values are mean ± SE. *p* < 0.05 are significant, *p < 0.05 versus Control, ^&^ *p* < 0.05 versus cMEL group.: Melphalan group, MEL+Chr50: Melphalan +Chrysin50 group, MEL+Chr75: Melphalan +Chrysin75 group, Chr50: Chrysin50 group, Chr75: Chrysin75 group.

Regarding microRNA analysis, levels of miR‐375 and miR‐204 were significantly elevated in the skin tissue of melphalan‐treated mice compared to the control group. However, therapeutic intervention with chrysin in both the Mel + Chr50 and Mel + Chr75 groups led to a significant downregulation of these microRNAs relative to the Mel group (Figure 6).

## 4 Discussion

This study demonstrates that melphalan disrupts skin and hair follicle homeostasis with concomitant upregulation of miR‐204 and miR‐375, suggesting their potential involvement in shifting balance from autophagy toward apoptosis, and that chrysin attenuates these microRNAs, correlating with protection against tissue damage. These findings identify a specific epigenetic mechanism underlying alkylating agent‐induced dermatological toxicity and suggest miR‐204/375 as potential therapeutic targets for preventing chemotherapy‐related tissue injury.

In this study, melphalan, an alkylating drug, increased the expression of apoptosis markers such as caspase‐3, BAX, BCL2, and BAX/BCL2 ratio while severely reducing autophagy markers like LC3β, Beclin‐1, and Atg5,7,12 in skin and hair follicle tissues. This outcome aligns with the known toxic effects of alkylating drugs, which alkylate nucleic acids and inhibit DNA synthesis, often leading to increased apoptosis (van den Boogaard et al. 2022) and decreased autophagy (Lin et al. 2023) in healthy tissues. After melphalan treatment, increased expression of miR‐204 and miR‐375 was detected in skin and hair. Although these microRNAs have not been extensively studied in skin and hair tissues, previous research showed their increased expression following alkylating agent exposure in tissues like the hippocampus (Ebadi et al. 2025). In this study, their upregulation was linked to increased apoptosis markers and suppressed autophagy markers. Several studies indicate that miR‐204 and miR‐375 exert their regulatory effects by promoting apoptosis. Furthermore, LC3, a key marker of autophagy, is inhibited by miR‐204 (Feng et al. 2020), and the expression of atg7 and LC3 genes are direct targets of miR‐375 (Zhao et al. 2020). Due to the presence of epidermal stem cells in the hair follicle protrusions and the basal layer of the epidermis, skin and hair are among the fastest dividing cell populations in the body (Morgun and Vorotelyak 2020). Skin keratinocytes (from the basal layer to the stratum corneum) and hair follicles (in all three phases—anagen, catagen, and telogen) undergo tightly regulated proliferation and differentiation processes. Continuous molecular interactions in the skin manage proliferation, differentiation, and apoptosis (Al‐Dhubaibi et al. 2025). The findings of this study showed that melphalan adversely affects skin and hair tissue, evident by reduced epidermal height and hair follicles in the anagen phase, indicating an imbalance between proliferation and cell death. These histopathological effects are likely mediated by the regulatory microRNAs miR‐204 and miR‐375 targeting autophagy and apoptosis pathways. Recent research indicates that abnormal microRNA expression in body tissues can be effectively modulated by flavonoids (Adinew et al. 2021). Flavonoids influence miRNAs either through direct binding or by interacting with associated effector proteins, thus playing a vital role in regulating proliferation, apoptosis, and metastasis (Proença et al. 2023). Among natural flavonoids, chrysin shows promising potential in modulating apoptosis, autophagy, and inflammation by affecting regulatory microenvironments (Kurkiewicz et al. 2025). The results showed that chrysin was associated with attenuation of melphalan‐induced histopathological damage. Specifically, the 75 mg dose was significantly associated with inhibition of apoptosis markers, increased epidermal thickness, and an improved anagen‐to‐catagen hair follicle ratio. Although the 50 mg dose was less effective in suppressing apoptosis and related tissue damage, it was still associated with increased autophagy, similar to that observed with the higher dose. The increase in the expression of autophagy markers following chrysin treatment indicates that skin and hair tissue cells are strongly trying to remove damaged and oxidized organelles against melphalan‐induced stress to ensure cell survival. in this study, the non‐significant trends in Mel + Chr50 likely reflect suboptimal dosing relative to chrysin's poor bioavailability and melphalan's overwhelming apoptotic signaling. At 50 mg/kg, chrysin may achieve partial miR‐204/375 suppression sufficient to enhance autophagy but insufficient to significantly reduce apoptosis or restore epidermal thickness. Optimization of chrysin delivery (e.g., Nano formulations) or extended treatment duration may enhance efficacy at lower doses. Notably, autophagy improvement at both doses suggests differential sensitivity of autophagy versus apoptosis pathways to miRNA modulation. An important consideration for translational relevance is the dose and oral bioavailability of chrysin. Consistent with most dietary flavonoids, chrysin has a reported oral bioavailability of approximately 1% in humans. The 50 and 75 mg/kg/day doses used in this study are standard for preclinical chrysin research, and allometric scaling indicates that the 75 mg/kg rat dose corresponds to a human equivalent dose of approximately 6 mg/kg/day, or 420 mg per day for a 70 kg adult. This dose is well within the range of chrysin doses commonly used in over‐the‐counter nutraceutical supplements (Gao et al. 2021). These findings therefore suggest that biologically plausible and readily achievable doses of this common dietary bioactive may protect against melphalan‐induced skin and hair damage. Overall, these findings indicate that chrysin can be considered a potential nutraceutical candidate or a supportive dietary intervention to help mitigate the side effects of alkylating agents such as melphalan.

In this study, limitations include the murine model, single time‐point analysis, and unconfirmed direct miRNA–mRNA interactions. Additionally, the study did not evaluate the LC3‐II/I ratio, which represents a more reliable indicator of autophagy activity, nor did it assess protein expression levels to confirm pathway activation at the translational level. Future work should employ skin organoids with miRNA mimics/antagomiRs, evaluate chrysin nanoformulations for improved bioavailability, and assess tumor efficacy interactions. Clinical translation requires Phase I trials of topical chrysin in chemotherapy patients with miR‐204/375 as predictive biomarkers.

## 5 Conclusion

Melphalan induces significant skin and hair follicle damage by disrupting the balance between apoptosis and autophagy, leading to epidermal thinning, impaired hair follicle cycling, upregulation of pro‐apoptotic markers, suppression of autophagy‐related genes, and increased expression of miR‐204 and miR‐375. These microRNAs appear to play a key role in mediating melphalan‐induced cytotoxicity. Co‐administration of the flavonoid chrysin, particularly at 75 mg/kg, markedly attenuated these effects by restoring epidermal thickness, improving hair follicle cycling, reducing apoptotic signaling, enhancing autophagy, and downregulating miR‐204 and miR‐375 expression. Overall, the findings suggest that chrysin protects skin and hair tissues against melphalan‐induced injury through modulation of miRNA‐regulated apoptosis and autophagy pathways, highlighting its potential as a supportive dietary intervention and nutraceutical candidate to reduce chemotherapy‐associated dermatological side effects.

## Author Contributions

**Zahra Parsamanesh:** validation, software. **Malihe Soltani:** supervision, writing – review and editing, project administration, formal analysis. **Negar Shafaei‐Bajestani:** writing – original draft, data curation. **Maryam Moghimian:** methodology, conceptualization, resources. **Zahra Rajabzadeh:** visualization, investigation.

## Funding

The authors have nothing to report.

## Ethics Statement

All experimental procedures were approved by the Ethics Committee of the Ministry of Health and Medical Education of the Islamic Republic of Iran (Approval No: IR.GMU.REC. 1402.004; Date: 2024‐01‐30). All efforts were made to minimize animal suffering throughout the study. Also, this study adheres to internationally accepted standards for animal research, following the 3Rs principle. The ARRIVE guidelines were employed for reporting experiments involving live animals, promoting ethical research practices.

## Conflicts of Interest

The authors declare no conflicts of interest.

## Data Availability

The data that support the findings of this study are available on request from the corresponding author. The data are not publicly available due to privacy or ethical restrictions.
